# Prediction of Actual from Climatic Precipitation with Data Collected from Northern Poland: A Statistical Approach

**DOI:** 10.3390/s23031159

**Published:** 2023-01-19

**Authors:** Jacek Barańczuk, Martina Zeleňáková, Hany F. Abd-Elhamid, Katarzyna Barańczuk, Salem S. Gharbia, Peter Blišťan, Cécil J. W. Meulenberg, Peter Kumer, Włodzimierz Golus, Maciej Markowski

**Affiliations:** 1 Coastal City Living Lab, Faculty of Oceanography and Geography, University of Gdansk, 80 309 Gdańsk, Poland; 2 Institute of Circular and Sustainable Construction, Faculty of Civil Engineering, Technical University of Košice, 042 00 Košice, Slovakia; 3 Department of Water and Water Structures Engineering, Faculty of Engineering, Zagazig University, Zagazig 44519, Egypt; 4 Department of Environmental Engineering, Faculty of Civil Engineering, Technical University of Košice, 042 00 Košice, Slovakia; 5 Centre for Environmental Research Innovation and Sustainability CERIS, Department of Environmental Science, Atlantic Technological University, F91 YW50 Sligo, Ireland; 6 Institute of Geodesy, Cartography and Geographical Information Systems, Faculty of Mining, Ecology, Process Control and Geotechnologies, Technical University of Košice, 042 00 Košice, Slovakia; 7 Mediterranean Institute for Environmental Studies, Science and Research Centre Koper, 6000 Koper, Slovenia

**Keywords:** actual precipitation, climatic precipitation, precipitation, prediction, Poland

## Abstract

Water is a basic element of the natural environment and the most important component in human water management. Rainfall is the main source of water. Therefore, determining the amount of precipitation reaching the ground using sensors is crucial information. Precise precipitation data are necessary for better modeling quality, as the observation data from weather stations are used as basics for weather model assessment. The authors compared precipitation from the Hellmann rain gauge (climatic precipitation, 1.0 m above the ground surface) measured throughout the year and the GGI 3000 rain gauge (actual precipitation on the ground level) measured from April to October. Measurement sequences from the years 2011–2020 were considered. The data for analysis were obtained from a weather station located in northern Poland. The authors analyzed the relationships between data from the two sensors. A comparative study showed that the measurements of actual precipitation are higher and there are strong relationships between actual and climatic rainfall (r = 0.99). Using the introduced coefficient it is possible to determine the full–year actual precipitation with high probability, taking into account the precipitation with a correction from the winter half-year and the actual precipitation from the summer half-year, which is of great importance in the calculation of the water balance.

## 1. Introduction

Water is a basic component of the natural environment, so it has a huge impact on the functioning and development of the economy, as well as everyday life. Therefore, water is a basic indicator that could help in describing global climate change. One of the basic elements of the water cycle is precipitation, the amount of which is one of the most characteristic manifestations of the annual variability of the natural environment [1,2]. The occurrence of precipitation, i.e., its formation and disappearance, is characterized by certain parameters during the year and on a multi-year scale [3,4]. Precipitation can take different forms depending on the temperature, and most often occurs in the form of rain [5,6], snow [7,8,9], and much less often and only under appropriate conditions in the form of ice [10,11,12].

Due to the rapid climate change caused by human activity, rainfall can take the form of violent events in the form of, among others, heavy rain [13,14]. During these extreme phenomena, climatic precipitation can be recorded in bulk over a longer period (mostly weeks). However, classic measuring instruments are not adapted to large rainfall in a short time, and thus the exact amount of daily precipitation recorded through them often remains inaccurate. Hence, there is a strong need to determine the exact amount of precipitation for a short time interval such as knowledge of daily precipitation (even though heavy downpours). It allows the proper design of stormwater and sewage systems and determination of the gain of water in the catchments, which allows effective prediction of floods and local flooding and gives the opportunity to store as much precipitation as possible and save it in the system for the period of water shortage. Thus, knowing the actual and precise amount of precipitation allows us to fight both droughts and floods.

Pluviometers are the most commonly used instruments to record precipitation [15] and comprise non–registering pluviometers, tipping bucket rain gauges, and self–registering pluviometers [16,17]. A pluviometer is usually an open, cylindrical container with vertical sidewalls. However, studies have shown that pluviometers installed above the ground do not capture all precipitation reaching the ground surface [18,19]: systematic losses vary according to the type of precipitation (snow, sleet, or rain) and the wind speed [20]. For example, according to Sieck, Chang, and Harrison, pluviometers installed on a ground level show higher precipitation measurements than those installed above ground [21,22]. Therefore, for many hydrological data, it is necessary to first correct the data and include measurement errors [21] before performing the analysis and applications. It turns out that the corrections are not precise or may even increase the error [22]. For this reason, the original data should always be archived as a base information source. Variations of space and time in precipitation falls have significant implications for the so-called earth systems. These variations can often be linked to biases in precipitation amounts [23,24], especially in high-resolution models [25]. Consequently, it is extremely important to maintain the continuity of data collection, and these data serve as the most valuable basis for future model improvements.

Additionally, since different sizes of pluviometers are used in different countries, the measurements are not precisely comparable to other devices [26]. However, some guidelines can be followed to standardize precipitation measurements. Thus, the design requirements of any pluviometers are such that they must be placed on flat ground to avoid the possibility of runoff disturbance; the aperture of the instrument shall be above the expected maximum snow depth and above the potential height of any soil splash erosion. Hence, in over 100 countries, pluviometers are installed at a height of between 0.5 m and 1.5 m [26].

In Poland, the most common method for determining precipitation used by the Institute of Meteorology and Water Management (IMGW–PIB) is measurement with a Hellmann rain gauge installed at a level 1.0 m above the ground surface [16,17], while precipitation measured on the ground level is collected with the GGI3000 rain gauge only at some weather stations. Additionally, in the IMGW–PIB weather station network, telemetric precipitation measurements are made with a 10-min time resolution. Telemetric data are collected all year round, also during winter.

Precipitation measured with a Hellmann rain meter is defined as climatic precipitation measured at 1.0 m above the ground surface, and precipitation measured with the GGI 3000 rain gauge is defined as actual precipitation on the ground surface [18]. Unfortunately, in Poland, actual precipitation is measured only in the summer half-year, while climatic precipitation is measured throughout the year. The use of two types of instruments and the use of two different types of precipitation means that there may be differences in the measurement results in the same place and at the same time. In the Hellmann rain gauge, the rain falls obliquely in the wind and causes inaccuracies in the measurement. It does not receive all the precipitation reaching the ground. In the case of the GGI 3000 rain gauge located on the ground, it eliminates disturbances from the wind, which is the biggest disturbing factor [17,18].

The authors therefore decided to answer questions in this study including the following: How is the relationship between climatic precipitation and actual precipitation shaped? What is the interdependence between them during the year? What is the variability of both quantities, and is it possible to extrapolate the obtained affinities to the winter half-year? To answer these questions, data were collected and analyzed from both climatic precipitation and actual precipitation at the same location near Gdańsk.

The water balance or modeling in Poland is based on precipitation, and the developed formula would reduce this underestimation, which is an important issue in the management of water resources and has a practical dimension, e.g., when constructing infrastructure: bridges, culverts, or sewage systems.

## 2. Materials

### 2.1. Study Area

**Figure 1:**
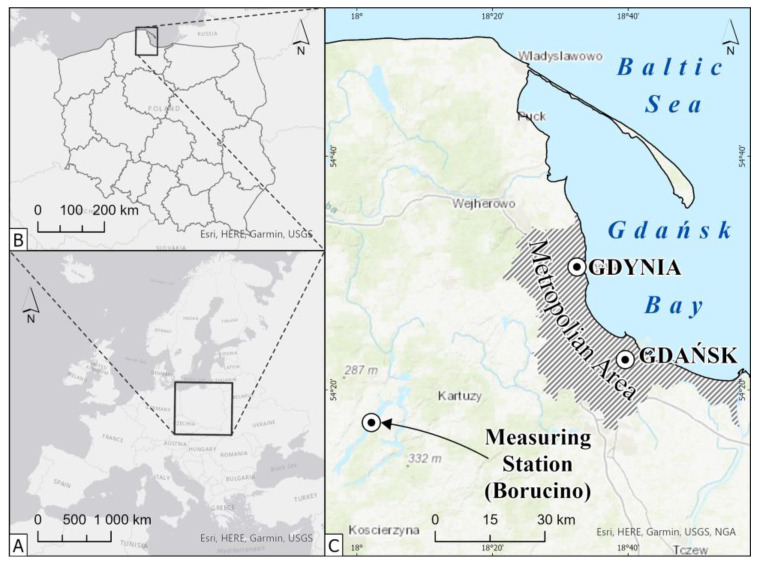
Location map of the study area in the context of (**A**)—Europe, (**B**)—Polish voivodeships, (**C**)—Tri-City Metropolitan Area.

The measuring/climatological station included in the land-based network of the IMGW-PIB in Warsaw is located at the Limnological Station of the Institute of Geography of the University of Gdańsk. The station is located a short distance from Gdańsk, where the University of Gdańsk and the Coastal City Living Lab (CCLL) are located. It is one of the closest weather stations to CCLL, where such analyses can be performed. It is located in the eastern part of the Kashubian Lake District in northern Poland, and the first measurements of basic meteorological parameters were made in 1961. The location of the weather station area (54°15′12.16″ N, 17°59′27.83″ E) and its height (163.0 m asl) have been unchanged from the beginning of its establishment (see Figure 1). The station’s functioning, including measuring atmospheric conditions and weather instruments, qualifies it to rank among the third-order meteorological stations with a barometer, i.e., climatological stations that perform, among other things, atmospheric pressure measurements [27].

Borucino weather station is located in the Polish Lowlands, west of the Vistula, within the Kashubian Lake District, in the catchment of Górna Radunia, which is a tributary of the Motława River (see Figure 1). It is a reference point and necessary help in determining the basic meteorological parameters for the lakelands of northern Poland [28] and for nearby Gdańsk. Its characteristic feature is its location on the northern shore of Lake Raduńskie Górne with an area of 387.2 ha and a depth of 43 m [29]. The weather station uses its location for measuring the evaporation rate from the lake. It makes the station one of the few Polish weather stations that has an evaporation buoy on the lake. Morphologically, the station is located within the post-glacial landscape. The landscape is shaped by the moraine plateaus, and individual moraine peaks reach (210 m asl) and (200 m asl) on the opposite side of the lake.

The meteorological station selected for the study is located in the area of the Central European Lowland. Lowland areas cover nearly 73% (Central European Lowland and East Baltic–Belarusian Lowlands) of Poland and 72% of Europe. This area only in a few places exceeds the height of 200 m asl. Climatically, the Central European Plain is under the predominant influence of oceanic air masses. The average annual precipitation amounts are in the range of 450–700 mm. [28]. The station is located relatively close to Gdańsk, which is the largest and most important city in northern Poland located on the Baltic Sea, which is part of the Tri-City agglomeration inhabited by over 1.6 million people (as of 2021). Therefore, the selected meteorological station can be treated as a station located in a representative area for analyzing actual and climatic precipitation.

### 2.2. Data Collection

Archival meteorological measurements of climatic and actual precipitation from the multi-year period from 2011 to 2020 were used. Precipitation data were obtained by an observer from measuring devices in accordance with IMGW–PIB guidelines. The data included precipitation measured with the GGI 3000 pluviometer (actual precipitation) and Hellmann rain gauge (climatic precipitation) in the summer half-year from May to the end of November, while the Hellmann rain gauge also provided data from November to the end of April for the winter half of the year, in order to extrapolate the actual precipitation.

## 3. Methods

### 3.1. Sensors

**Figure 2:**
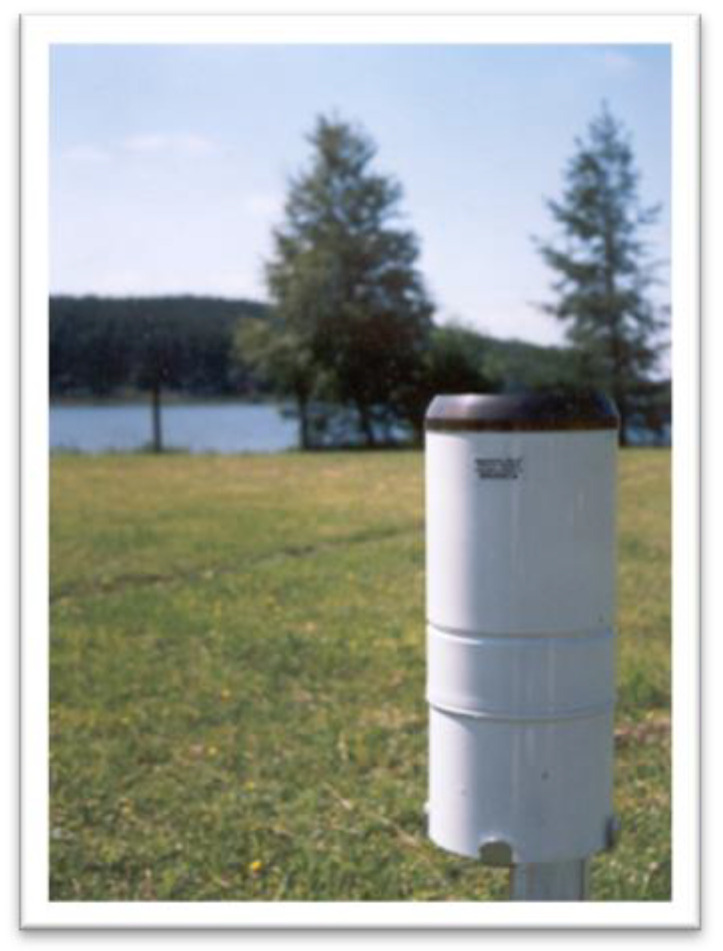
Hellmann rain gauge at the Limnological Station in Borucino.

The Hellmann rain gauge used to measure climatic precipitation is shown in Figure 2.

The instrument consists of three main parts: The top part is a metal cylinder, exactly finished, with a sharp, upper edge (usually made of brass) and a funnel in its lower part. Precipitation is collected by the upper, carefully made edge of the cylinder with an area of 200 cm^2^ and flows through a funnel into a tank located in the base of the rain gauge. During winters it accumulates snow in the upper part of the cylinder above the funnel. The base is a metal cylinder with a flat bottom, which, by placing a roller with a funnel on it from the top, connects the parts of the rain gauge. This construction of the rain gauge greatly reduces the evaporation of rainwater that collects in it during the day. The rain gauge is placed on a pole attached to a post 90 cm high above the ground in such a way that the rainfall collecting surface is placed horizontally at a height of 1.0 m above the ground surface. The accuracy of the instrument is 0.1 mm [30]. The precipitation is recorded once a day at 7.00 a.m.

**Figure 3:**
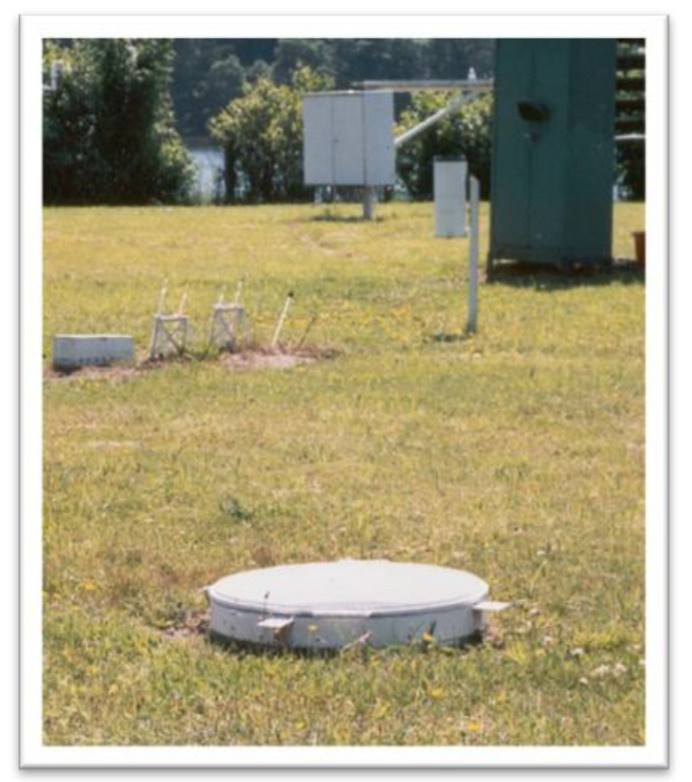
GGI 3000 evaporometer (foreground) at the Limnological Station in Borucino. Hellmann rain gauge can be seen in the back.

The GGI 3000 rain gauge, which is part of the GGI 3000 evaporometer set (see Figure 3), is used to measure the actual precipitation. The rain gauge is positioned so that its 3000 cm^2^ receptor is located 5 cm above ground level. The accuracy of the instrument is 0.1 mm [31]. The records are made from the beginning of May to the end of October, two times a day at 7.00 a.m. and 7.00 p.m. (CET).

### 3.2. Statistical Comparison of the Data Sets

The data from direct observations were saved digitally and copied to a spreadsheet. During the data entry, the precipitation of less than 0.0 mm was omitted due to the fact that the program also automatically entered 0.0 in the place of dry days, which caused errors in the calculations. Moreover, differences in the hours of reporting precipitation from rain gauges caused the authors to introduce an additional correction. In order to unify the obtained data, the daily precipitation readings at 7.00 a.m. were used.

Quantitative analysis of the obtained data was performed in a spreadsheet. The datasets were statistically analyzed [32,33,34,35], and statistics were used to carry out the calculations. The obtained daily climatic precipitation data were compared with the daily actual precipitation data.

The final calculations consisted of the extrapolation of monthly totals of actual precipitation for the winter half of the year. In order to extrapolate the data for the winter half of the year, the Formula (1) proposed by the authors of this work was used:

(1) $$P_{MG}=\left(P_{MH}+a\right)b^{n}$$

where:

*P_MG_* is the actual precipitation (GGI 3000),

*P_MH_* is the climatic precipitation (Hellmann),

*a*, *b*, *n* are empirical coefficients.

## 4. Results

### 4.1. Measures of Precipitation Variability

**Figure 4:**
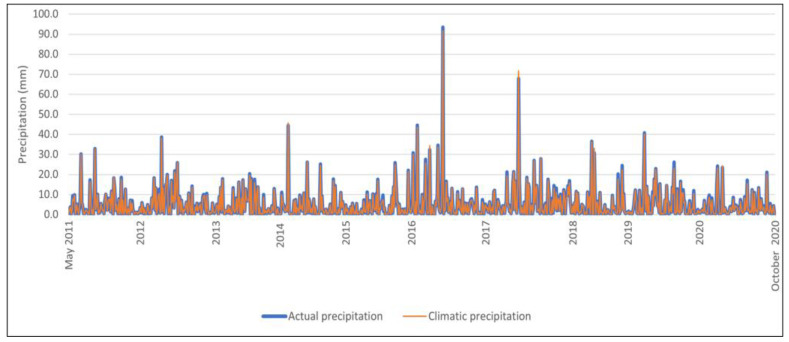
Daily actual precipitation and climatic precipitation from May to October in the period from 2011 to 2020.

In the study period from 2011 to 2020, actual and climatic precipitation was measured on 860 days. Figure 4 shows the variability of daily actual and climatic precipitation. The maximum value for actual precipitation and climatic precipitation occurred on 14 July 2006. The actual precipitation was 93.4 mm, and the climatic precipitation was 91.6 mm. The minimum actual and climatic precipitation was 0.1 mm and was recorded 46 times (per year). Using the position measure, the average actual precipitation ($\bar{x}$) was 5.0 mm and the average climatic precipitation was 4.8 mm.

The most common actual precipitation in the period under review (D) was 0.1 mm and occurred 46 times, while the most common climatic precipitation was 0.1 mm and 0.2 mm and occurred 45 times.

**Figure 5:**
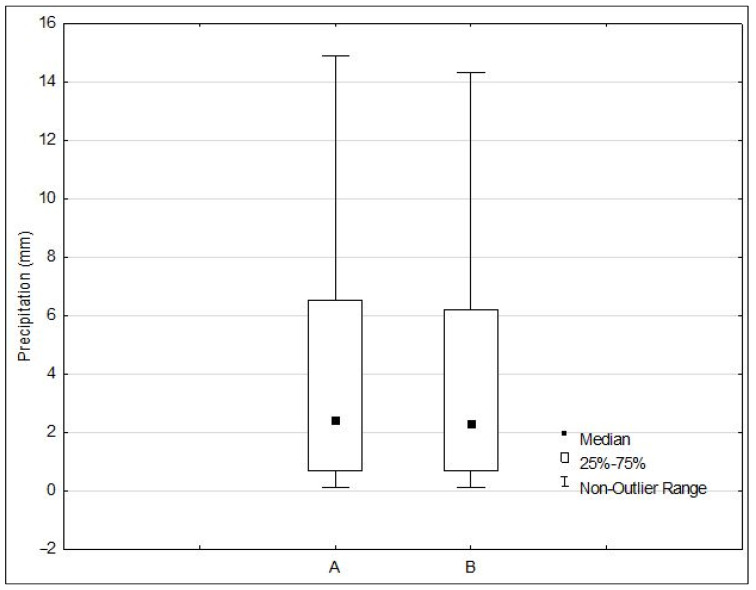
Mean Values of actual precipitation (**A**) and climatic precipitation (**B**).

During the study, 25% of days had actual precipitation of not more than 0.7 mm, and 75% of days had actual precipitation of not less than 0.7 mm. The first quartile (Q_1_) for precipitation is the same and equals 0.7 mm (see Figure 5). During the period under review, 75% of days had actual precipitation of not more than 6.5 mm, and 25% of days had actual precipitation of not less than 6.5 mm. In contrast, the third quartile (Q_3_) for daily climatic precipitation was smaller and reached 6.2 mm. Dispersion measures showed a fairly large variation in the daily values of actual and climatic precipitation. The variance (S^2^) for the actual precipitation was 54.5 mm, and for climatic precipitation 53.0 mm.

The daily actual precipitation differs from the arithmetic mean by an average of 7.4 mm, while the daily climatic precipitation differs from the arithmetic mean by an average of 7.3 mm. This amount is 148% for actual precipitation, and for climatic precipitation, 152% of the arithmetic mean [V(s)], which means that the variation in daily actual and climatic precipitation is very large. In the middle 50% of the dataset, the difference between the extreme values [R(Q)] of daily actual precipitation reached 5.8 mm, and for daily climatic precipitation 5.5 mm. The difference between the highest and lowest value (R) of the daily actual precipitation was 93.3 mm, while for daily climatic precipitation, the difference reached 91.5 mm.

### 4.2. Correlation of Actual and Climatic Precipitation during the Summer Half of the Year

**Figure 6:**
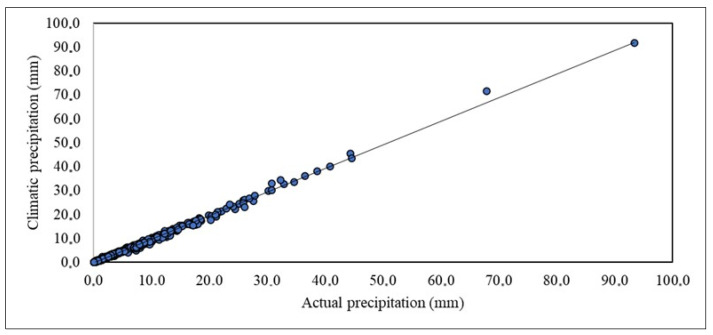
Relationship between the actual precipitation and climatic precipitation.

In order to determine the relationship between actual precipitation and climatic precipitation, a graphical method was used, the so-called correlation diagram presenting the values of both studied variables for daily sums and decade sums [32]. Based on Figure 6, it was found that there is a linear relationship between the studied variables, and when the relationship of the studied features is linear, the Pearson linear correlation coefficient (y = 0.9844x − 0.0738) is most often used to determine the measure of interdependence.

Considering the relationship between actual precipitation and climatic precipitation, the correlation coefficient is 0.998 (*p* < 0.05), which indicates a very strong relationship between the features. The very strong relationship between the features is also indicated by the coefficient of determination, which is very high and reached 0.996, which means that as much as 99.6% of the variability of daily climatic precipitation is explained by the formation of daily actual precipitation. Only 0.4% of daily climatic precipitation depends on other factors. A strong relationship in Figure 6, is particularly visible. The points are almost perfectly arranged along a straight line. It can be interpreted as meaning that the individual changes in actual precipitation correspond to almost all individual changes in climatic precipitation.

### 4.3. Extrapolation of Actual Precipitation to the Winter Half-Year and Comparison with Climatic Precipitation from 2011 to 2020

Based on the analysis of the variability of actual and climatic precipitation, the extrapolation of actual precipitation to the winter half of the year was carried out. The analysis of variability performed using correlation and regression showed a very strong relationship between actual and climatic precipitation. As much as 99% of climatic precipitation is associated with actual precipitation. Extrapolation of actual precipitation to the winter half-year is carried out using climatic precipitation measured with a Hellmann rain gauge, and the Formula (2) is developed:

(2) $$P_{MG}=\left(P_{MH}+0.0738\right)0.9844^{-1}$$

This formula, proposed by the authors of this work in general research methods, is used to extrapolate actual precipitation using climatic precipitation. The extrapolation shows that the actual precipitation is higher than the climatic precipitation in the winter half-year. Climatic precipitation accounts for 96% of actual precipitation. The average actual precipitation in the winter half of the year was 310.0 mm, and the climatic precipitation was 297.9 mm.

**Figure 7:**
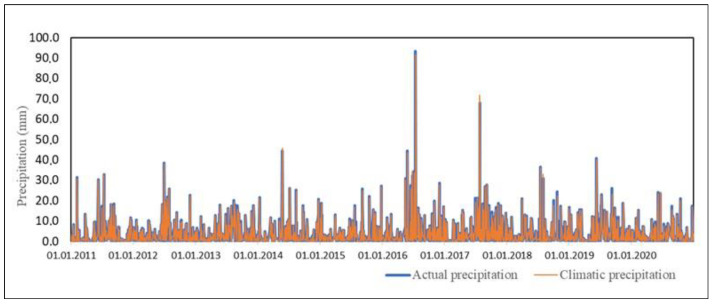
Distribution of the actual precipitation and climatic precipitation in the period from 2011 to 2020.

The extrapolation of actual precipitation to the winter half-year allows us to compare the results with the summer half-year and obtain the results of actual precipitation for the period from 2011 to 2020 (see Figure 7). In the period 2011 to 2020, the actual precipitation reached 7393.0 mm, which is greater than the climatic precipitation (7141.0 mm). On average, the value of actual precipitation per year is 739.3 mm, and that of climatic precipitation is 714.1 mm. The average annual climatic precipitation is lower by 25.2 mm than the actual precipitation. Climatic precipitation accounts for 96.6% of actual precipitation.

## 5. Discussion

The height of the rain gauge installation above ground has been the subject of analysis and discussion for over a century. This is particularly important for hydrologists and limnologists that calculate water balance. The amount of precipitation that falls on the ground is important for the obtained results. Knowledge of precipitation amounts is particularly important in hydrological studies, e.g., runoff from the entire catchment, where large physiographic areas are often involved. With a high degree of probability, it can be concluded that the failures of some water constructions and flood damages occurring in recent years were caused by improper amounts of rainwater adopted in the calculations. The knowledge of the value of precipitation is also important for melioration. It is very important to determine the diameters of the drains, determine the depth of the drainage ditches, and design regulatory water constructions. This also applies to irrigation, where a close relationship between the amount of precipitation and water demand can be observed [19].

The amount of precipitation recorded is also very important in limnological research. It allows us to properly use natural storage reservoirs such as lakes in the fight against floods or droughts. Due to the fact that reservoirs store water, during violent or prolonged downpours, they hold surplus water and flatten the flood wave. However, during drought, water from retention reservoirs feeds the river network, and drought is less severe, especially for agriculture.

A very important role is also played by the rainfall measurement technique, the specific application of which has caused most of the still insurmountable difficulties. The WMO has attempted to carry out comparative measurements, at the same time proposing a model of a reference rain gauge with a reception area of 127 cm^2^. Pocelet and Rusyn [18] carried out a comparative series model of particular importance, in which they included Hellmann rain gauges, the most commonly used pluviometers in Europe, America, and Asia, as well as the Weather Bureau of Great Britain with a Nipher shield, and Tretyakov and Swedish gauges with wind shields. The published results did not conclude that any of the instruments were superior; all of them allow the assessment of the height of precipitation with a large error, depending on both meteorological conditions and the setting of the instrument [18].

When measuring precipitation, there are also losses associated with evaporation and wetting of the rain gauge. In general, the best results are obtained with a glossy white rain gauge. The issue of precipitation loss is also related to the loss of water associated with wetting the walls of the rain gauge. It should be noted that small precipitation (0.1 mm) is immeasurable, and is not included in datasets, which further increases the size of losses. In addition, the measurement of precipitation at some altitude is associated with air turbulence, and thus affects the measurement result. A simple and proven solution is to measure climatic precipitation, which eliminates aerodynamic error, and greatly reduces evaporation and wetting error [18].

Sieck and others [21] showed that in the Mississippi basin rain gauges exposed to wind show 2% to 10% less rain than those placed at ground level. They also cited that measuring instruments should be situated away from any obstacles to avoid wind disturbance. The required distance from the obstacles should be at least 4 but preferably 10 times away in relation to the instrument height [21,22]. The differences resulting from the height of the measurement, and thus the influence of wind on the measurement, were shown by such researchers as Molga, Kuźniar, and Chomicz [36,37,38]. The difference between actual precipitation and climatic precipitation was obtained by Kuźniar [37] on the basis of his research carried out at the Institute of Soil Science and Plant Cultivation—State Research Institute in Puławy and at the Higher Agricultural School Experimental Station in Ostrów Szlachecki (1957–1961) [37]. Actual precipitation was 6% higher than climatic precipitation. Chomicz [39] carried out comparative measurements at the National Hydrological and Meteorological Institute station in Warsaw in 1963, where the actual precipitation was 5% higher than the climatic precipitation. The coincidence of the results obtained in completely different field conditions and at different times, and thus probably in different weather conditions, would indicate that the obtained calculations correspond to the natural amounts [39,40]. Similar calculations of actual precipitation were obtained in our work (4%) and in the work of Pasela and Zawory [41], where actual precipitation measured during the growing season (from April to October) was 4% higher than climatic precipitation sums measured.

Some sources also include information about differences in precipitation reaching 10% and more [42,43,44,45,46,47]. Molga [36] obtained similar results. In his research, the actual precipitation is 13.5% higher than the observed climatic precipitation. The research was carried out at the Agrometeorological Station in Brwinów in 1950 [26]. Chomicz [40] later also obtained other results. By analyzing the actual precipitation in Poland, he concluded that the values of actual precipitation can be up to 10 to 30% higher than the values of climatic precipitation. These differences vary depending on the location of the station and the season [38,40]. A similar relationship was obtained by Koschmieder [20], however, in mountainous regions of Poland. Research conducted in 1947–1958 showed that in this region, the average wind speed on the highest peak of Śnieżka (Sudeten) for the period of 12 years was 12 m/s. As a result, the measured precipitation was 65% of the actual precipitation, which corresponds to an increase in actual precipitation by 35% [20]. From the above cases, it can be seen that the actual precipitation is always greater than the climatic precipitation. The main reason that actual precipitation is sometimes much greater than climatic precipitation is that measuring precipitation on the ground eliminates aerodynamic error completely [20,42].

As with any research, there are uncertainties in the study. The question arises whether the differences between the types of rainfall would be more difficult to estimate or have a greater statistical error if the relationship between actual precipitation and climatic precipitation is much weaker, e.g., r = 0.8. Of course, the accuracy of the measurement may be affected by extreme weather events interfering with the data results, such as storms or tornadoes in the USA. However, here in the analyzed area, such phenomena occur extremely rarely or not at all, they do not have a major impact on the research results. An interesting issue that could be considered would also be to outline the absolute difference between actual and climatic precipitation for the months in the period 2011–2020 and use these differences to calculate a coefficient that makes actual precipitation realistic. The aim of future research could be to perform a comparative test of the GGI 3000 rain gauge with instruments located at different heights above the ground, e.g., the Vaisala WXT510 automatic station and the THIES CLIMA laser sensor to show differences or lack thereof. On the basis of these studies [48,49,50,51,52,53], the implementation of comparative coefficients would bring new information for calculating the water balance.

## 6. Conclusions

In this paper, the authors presented a problem related to the measurement of precipitation using different rain gauges. The study was about analyzing precipitation in the period from 2011 to 2020. The data came from the weather station located in the Limnological Station of the University of Gdańsk in Borucino. The authors focused on relationships between the Hellmann rain gauge and the GGI 3000 rain gauge.

According to the authors, the analysis carried out and the interpretation of the results obtained answers affirmatively to the question asked in the introduction. The presented analysis regarding the possibility of using a formula extrapolating the results obtained from the measurement of actual precipitation for the winter half of the year on the basis of climatic precipitation data for the winter period in order to determine the variability of actual precipitation throughout the year was correct. This indicates a high convergence of the distribution of actual and climatic precipitation n other environmental parameters. With the help of the introduced formula/correction coefficient, it is possible to extrapolate with a high probability the year–round actual precipitation by taking into account precipitation with a correction from the winter half of the year and actual precipitation from the summer half-year.This result is of great importance in the entire water management and in particular in the calculation of the water balance because it clarifies the precipitation data. The outcomes of this study could help with creating future plans for water resource management in different countries.

## Data Availability

Not applicable.
